# Acute hospital dementia care: results from a national audit

**DOI:** 10.1186/s12877-016-0293-3

**Published:** 2016-05-31

**Authors:** Suzanne Timmons, Emma O’Shea, Desmond O’Neill, Paul Gallagher, Anna de Siún, Denise McArdle, Patricia Gibbons, Sean Kennelly

**Affiliations:** Centre for Gerontology and Rehabilitation, School of Medicine, University College Cork, The Bungalow, Block 13, St. Finbarr’s Hospital, Douglas Road, Cork, Ireland; Centre for Ageing, Neuroscience and the Humanities, Trinity Centre for Health Sciences, Tallaght Hospital, Dublin, Ireland; Quality and Patient Safety Audit Services, Health Service Executive, Dublin, Ireland

**Keywords:** Dementia, Quality of care, Hospitals, Audit, Assessment, Antipsychotics, Discharge planning, Specialist services, Staff training

## Abstract

**Background:**

Admission to an acute hospital can be distressing and disorientating for a person with dementia, and is associated with decline in cognitive and functional ability. The objective of this audit was to assess the quality of dementia care in acute hospitals in the Republic of Ireland.

**Methods:**

Across all 35 acute public hospitals, data was collected on care from admission through discharge using a retrospective chart review (*n* = 660), hospital organisation interview with senior management (*n* = 35), and ward level organisation interview with ward managers (*n* = 76). Inclusion criteria included a diagnosis of dementia, and a length of stay greater than 5 days.

**Results:**

Most patients received physical assessments, including mobility (89 %), continence (84 %) and pressure sore risk (87 %); however assessment of pain (75 %), and particularly functioning (36 %) was poor. Assessment for cognition (43 %) and delirium (30 %) was inadequate. Most wards have access at least 5 days per week to Liaison Psychiatry (93 %), Geriatric Medicine (84 %), Occupational Therapy (79 %), Speech & Language (81 %), Physiotherapy (99 %), and Palliative Care (89 %) Access to Psychology (9 %), Social Work (53 %), and Continence services (34 %) is limited. Dementia awareness training is provided on induction in only 2 hospitals, and almost half of hospitals did not offer dementia training to doctors (45 %) or nurses (48 %) in the previous 12 months. Staff cover could not be provided on 62 % of wards for attending dementia training. Most wards (84 %) had no dementia champion to guide best practice in care. Discharge planning was not initiated within 24 h of admission in 72 % of cases, less than 40 % had a single plan for discharge recorded, and 33 % of carers received no needs assessment prior to discharge. Length of stay was significantly greater for new discharges to residential care (*p* < .001).

**Conclusion:**

Dementia care relating to assessment, access to certain specialist services, staffing levels, training and support, and discharge planning is sub-optimal, which may increase the risk of adverse patient outcomes and the cost of acute care. Areas of good practice are also highlighted.

## Background

Dementia is characterized by progressive impairment in domains such as memory, orientation, comprehension, language, and judgement and can also affect personality, mood and behaviour. In Ireland, approximately 41,700 people were estimated to have dementia as of 2006, and this figure is projected to rise to between 140,580 and 147,000 by 2041 [1], making it a significant public health issue. According to the World Health Organisation [2], the rapidly increasing prevalence of dementia will place unprecedented demands on health care services, challenging governments to develop and improve the services providing care for people with dementia, in order to meet these demands.

Older people with dementia on average have three or more physical comorbidities, which at some point necessitate hospital admission [3] and the prevalence of dementia in hospital ranges from 29-42 % in adults over 70 [4, 5]. Hospital admission is distressing for this population and is associated with cognitive and functional decline [6], greater institutionalisation post-discharge [7, 8], and higher mortality rates [5, 7].

Acute hospitals are not currently equipped to provide best dementia care [9]. Patients are generally not hospitalized for the dementia itself [10] and therefore the dementia is rarely the care or treatment priority. Further, staff training and knowledge around dementia care can be poor [9, 11] and it often goes undetected in the acute setting [12]. This can result in unmet needs, and an increase in behavioural and non-cognitive symptoms of dementia (BPSD) [13], which staff report to be burdensome [14]. Other factors contributing to poor quality dementia care in hospitals include poor multidisciplinary assessment, [15, 16], under-diagnosis and treatment of pain [17], failure to collect collateral history [18], and the inappropriate prescription of antipsychotic drugs [19].

A diagnosis of dementia is also related to greater length of stay (LOS) in hospitals [20], and an increased cost of care. Dementia care is estimated to cost €21 million per annum in Ireland, based on the number of Hospital In-Patient Enquiry (HIPE)-coded discharges of people with a recorded formal diagnosis of dementia [1]. A more recent study has estimated that dementia-related admissions lead to approximately 246,908 additional days per annum in hospitals in Ireland, at an associated cost of €199 million [21]. Reasons cited for increased LOS are largely systemic, include poor discharge planning, delayed discharge relating to post-hospital care availability for complex conditions, and lack of communication and coordination between health service providers [22, 23].

It is clear from the above literature that improving dementia care in acute hospitals should be an urgent priority in Ireland. Countries including England and Wales [15, 16] have carried out audits of dementia care to gain a baseline picture of the quality of care provision in their hospitals. The objective of this audit is to assess, for the first time, the quality of dementia care in acute public hospitals in the Republic of Ireland.

## Method

As there are no standards in place for dementia care in acute hospitals in Ireland currently, this audit measured care against international best practice guidelines.

All 35 acute public hospitals admitting adults within the Health Service Executive (HSE) were included in this audit, and the data was collected between April and September 2013. The audit tools were developed for use in the National Audit of Dementia Care in General Hospitals in England and Wales [15], and with permission from the Healthcare Quality Improvement Partnership, minor alterations were made to the wording of questions, where necessary, so that they made sense in the context of the Irish healthcare system.

For this audit, (i) a retrospective chart review tool, as well as (ii) hospital level organisation (iii) ward level organisation (iv) and ward environment tools were employed. The results of the ward environment module of the audit are not reported here.

(i) Chart Review: This tool tracked the quality of care received by patients (*n* = 660) retrospectively, from admission through to death/discharge during a single admission, collecting information on demographics, assessments carried out on or during admission, referral to specialist services and discharge planning. Charts were identified using the HIPE system which collects national demographic, clinical and administrative data on discharges from, and deaths in all acute public hospitals. Inclusion criteria included a recorded HIPE diagnosis (primary or other) of dementia (International Classification of Disease (ICD)-10 codes: F00, F01, F02, F03, F05.1), a LOS greater than 5 days, and a hospital discharge date between 1^st^ September 2012 and 31^st^ January 2013.

The data was collected by the audit team as well as Specialist Registrar trainees in geriatric medicine, all of whom received comprehensive training in the use of the audit tool. Where possible, hospitals had their charts reviewed by independent auditors (76 %). Furthermore, 25 % of charts were independently re-reviewed by the audit team. The tool was found to have good inter-rater reliability and percentage agreement, similar to that found in the England & Wales audit [15].

(ii) Hospital Organisation: The audit team used this tool to collect data on hospitals’ (*n* = 35) policies, procedures, structures and guidelines relating to dementia care, through structured interview with senior hospital management and clinicians.
(iii) Ward Organisation: This tool collected data on staffing levels and supports, availability of specialist services, and systems in place to support people with dementia on wards through structured interview with the ward manager. In each hospital, 2-3 wards that admit adults were selected, with at least one medical and one surgical ward chosen per hospital. In total, 77 wards were audited by the audit team.

### Data analysis

Most of the data collected was categorical in nature, and valid percentages are reported in this study to describe this data. Median and interquartile ranges were used to describe the distribution of continuous variables, including age and LOS as the data did not have a normal distribution. For this same reason, the Mann-Whitney U-Test (non-parametric equivalent of the T-test) was used to investigate group differences in continuous variables. Data were analysed using SPSS version 20.

### Ethics

As this was an approved national audit, performed in partnership with the Health Service Executive’s National Quality and Patient Safety Directorate, we were exempt from seeking multiple hospital-level ethical committee approvals. Data collectors external to a hospital signed confidentiality agreements and access to charts at each site was coordinated through the hospital’s audit/quality department. Each hospital was assigned a code, kept securely by the audit coordinator, and individual hospital-level data was only released to that hospital’s Executive Officers. Similarly, each patient’s chart was assigned a code at source (to allow the re-audit of some charts for quality assurance) and no identifying patient details were recorded. The linking code was kept securely in the hospital’s audit/quality department and destroyed at the end of the audit.

## Results

### Demographics

Table 1 contains information about hospital size groupings as determined by bed capacity, as well as the number of geriatricians, hospitals and charts reviewed per group. Patient (*n* = 660) demographics relating to sex, age, place of admission, and ward type can be found in Table 2; few patients were treated according to an end of life care pathway or referred to specialist palliative care and a decision for resuscitation was documented in less than half of charts. In-hospital death occurred in less than 8 %. Of those who survived the admission, most were discharged to residential care or home, with 35 % of those originally admitted from home being newly institutionalised. The median LOS was 12 days (IQR = 7-28), however a Mann-Whitney U test revealed that LOS was significantly greater for those newly discharged to residential care (median = 35), than not (median = 10), *U* = 13024, *z* = -10.67, *p* < 0.001.

**Table 1:** Hospital characteristics and charts reviewed by bed capacity

Hospital size	80-150 beds	151-300 beds	301-600 beds	601-1000 beds
Number of geriatricians in the hospital	1-2	2-3	2-4	3-5
Number of hospitals	9	9	12	5
Number of Charts Reviewed	173	178	212	97

**Table 2:** Patient demographic information

Sex (% female, n)	61.8, 408
Age (Median, IQR)	83, 79-87
Admitted from home (%, n)	62.1, 410
Admitted from residential care (%, n)	32.7, 216
Admitted to Medical Ward (%, n)	66, 436
Admitted to Care of the Elderly Ward (%, n)	9.1, 60
Admitted to Surgical Ward (%, n)	8.8, 58
Treated on an end of life care pathway (%, n)	5.6, 37
Referred to Specialist Palliative Care (%, n)	6.7, 44
Decision recorded for resuscitation (%, n)	32.5, 215
In-hospital mortality (%, n)	7.7, 51
Discharged to residential care (%, n)	51.4, 339
Discharged home (%, n)	31.7, 209
New institutionalization post-discharge (%, n)	35, 144
Length of Stay (Median, IQR)	12, 7-28

### Multidisciplinary assessment

**Fig. 1:**
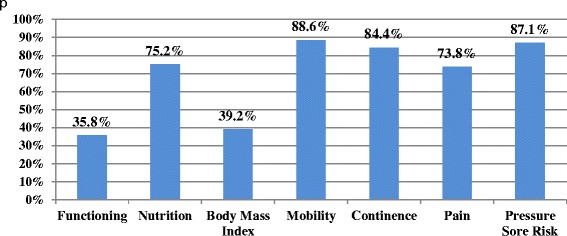
Physical Assessments Received by Patients with Dementia during Admission (*n* = 660)

In terms of physical assessment, this audit investigated whether or not patients received assessments of functioning (ADLs), nutrition, body mass index (BMI), mobility, continence, pain and pressure sore risk. As can be seen in Fig. 1, assessment of functioning and BMI was quite poor, with less than 40 % of patients receiving these assessments. Mobility, continence and pressure sore risk assessments were performed more regularly, with over 80 % of patients receiving these assessments. There is no record in almost one-quarter of the sample, of the person being asked about the presence of pain, or of any standardised pain assessment.

A gap was noted between hospital policy regarding physical assessments for people with dementia, and actual assessments carried out in practice. While 36 % of patients received a standardised assessment of functioning, 60 % of hospitals responded that it is policy that such an assessment should be carried out on all in-patients with dementia. Just over 75 % of patients received an assessment of nutrition; however 94 % of hospitals reported that all patients with dementia should receive this during an admission. Further, 39 % of patients had their BMI recorded, but 86 % of hospitals reported that this should always be recorded for patients with dementia.

**Fig. 2:**
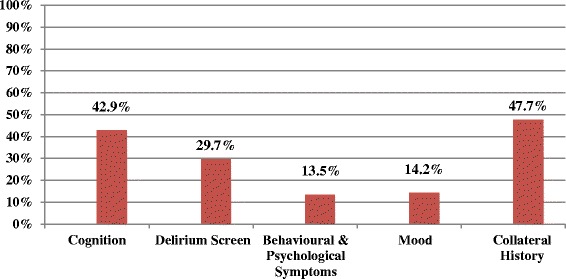
Mental Status Assessments Received by Patients with Dementia during Admission (*n* = 660)

This audit investigated whether assessments of cognition, delirium, BPSD and mood were carried out, and if collateral (informant) history was collected from a carer/relative. As seen in Fig. 2, less than half of patients received any of the mental assessments listed, with particularly low assessment of mood and BPSD, both at 14 %.

The frequency of physical and mental assessments completed was investigated by hospital size (bed capacity), and as seen in Table 3; the largest hospitals completed delirium screens, BPSD and mood assessments, and collected collateral history most often, while the smallest hospitals completed assessments of functioning, nutrition and cognition most often. However, large variation was observed between hospitals within each hospital size group (Table 3).

**Table 3:** Assessments completed (*n* = 660) by hospital size (Bed Capacity)

% (% range)	80-150 (*n* = 173)	151-300 (*n* = 178)	301-600 (*n* = 212)	601-1000 (*n* = 97)
Physical Assessment				
Functioning	44 (20-100)	41 (10-88)	36 (10-100)	15 (5-30)
Nutrition	83 (47-95)	80 (48-84)	72 (29-90)	66 (40-85)
BMI	66 (8-94)	47 (11-81)	44 (29-100)	46 (25-63)
Mobility	84 (50-100)	88 (71-100)	93 (76-100)	91 (75-100)
Continence	89 (73-100)	84 (52-100)	82 (20-100)	83 (50-100)
Pain	65 (10-100)	77 (40-95)	80 (55-100)	74 (60-90)
Pressure Sore Risk	84 (5-100)	93 (90-100)	84 (40-100)	88 (65-100)
Mental Assessment				
Cognition	50 (9-95)	45 (19-75)	38 (10-55)	39 (20-53)
Delirium Screen	23 (0-100)	18 (10-30)	37 (5-65)	48 (20-75)
Behavioural & Psychological Symptoms	9 (0-41)	10 (0-25)	14 (0-25)	28 (15-59)
Mood	15 (0-100)	10 (0-20)	14 (0-59)	23 (10-41)
Collateral History	29 (13-53)	34 (20-56)	62 (35-82)	74 (60-94)

Highlighting further disparity between policy and practice for mental assessment, 62.9 % of hospitals reported that all patients with dementia should receive a cognitive assessment, but only 42.9 % of patients actually had their cognition assessed during the admission. No collateral history regarding cognitive decline, time since the onset of memory problems, the nature of the disease progression, or loss of function, was collected for over half of patients (52.3 %), despite 62.9 % of hospitals reporting that a collateral history is collected for every patient with dementia.

### Specialist services

The majority of the 77 wards audited had access to specialist services at least 5 days per week, including Liaison Psychiatry, Geriatric Medicine, Occupational Therapy, Physiotherapy, Specialist Infection Control, and Specialist Palliative Care. Ward access to Continence Services and Social Work was poorer, and access to Psychology services is extremely limited (see Table 4). Further investigation into these services revealed that ward managers’ reports of availability differ across wards within the same hospitals e.g. 8.6 % of wards disagreed about the availability of Social Work and Psychology, and 17.1 % disagreed about Continence Services.

**Table 4:** Ward access to specialist services (*n* = 77)

Speciality service	No access	Limited^a^	Monday-Friday	Monday-Sunday
Liaison Psychiatry	4 %	3 %	42 %	51 %
Psychiatry of Old Age	26 %	3 %	58 %	13 %
Geriatric Medicine	16 %	-	52 %	32 %
Occupational Therapy	21 %	-	79 %	-
Social Work	47 %	-	52 %	1 %
Pharmacy	0 %	1 %	32 %	67 %
Physiotherapy	0 %	1 %	51 %	48 %
Dietetics	7 %	5 %	88 %	-
Speech & Language	9 %	10 %	81 %	-
Psychology	91 %	-	9 %	-
Infection Control	1 %	3 %	63 %	33 %
Tissue Viability	29 %	16 %	54 %	1 %
Continence	66 %	8 %	26 %	-
Palliative Care	3 %	8 %	21 %	68 %

^a^‘Limited’ access refers to 3 or less days per week

### Staffing training & support

**Fig. 3:**
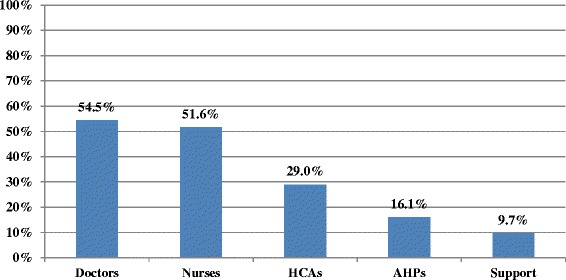
Dementia Awareness Training Provided to Staff by Hospitals in Previous 12 Months

A knowledge and training framework/strategy identifying necessary skill development for staff working with people with dementia was only in place in 21.2 % of hospitals. Only 2 hospitals included dementia awareness training on their staff induction programmes; however no hospital had mandatory dementia awareness training. Hospitals were asked if they had offered this dementia training to staff in the last 12 months (see Fig. 3), with just over half offering to nurses (51.6 %) and doctors (54.5 %). Other types of specific training in dementia were poorly provided by hospitals for both doctors and nurses respectively, in areas including capacity assessment (37.1 %, 20 %), hearing and visual impairment (8.6 %, 5.7 %), communication skills (5.7 %, 22.9 %), and behaviours that challenge (45.7 %, 57.1 %). The majority of ward managers (62.3 %) reported that cover could not be given for a staff member on the ward to attend training relating to dementia care.

There were registered nursing staff vacancies on 35 % of wards; however 69 % of wards had at least one vacancy between their permanent healthcare and nursing staff. The mean number of nursing vacancies on wards was 1.83 (SD = 2.13) with a minimum of 0 and a maximum of 9.

**Fig. 4:**
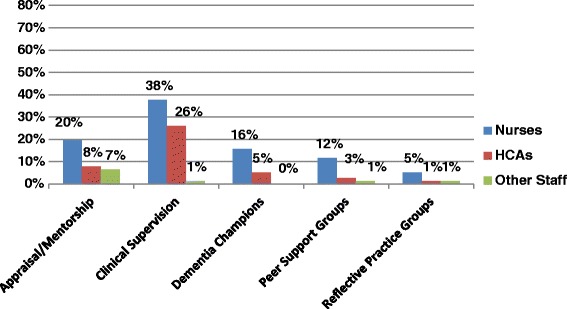
Systems in Place to Support Staff Caring for People with Dementia

The audit also investigated the types of supports available to staff on the wards caring for people with dementia (see Fig. 4), finding that appraisal and mentorship was not provided for nurses on 80 % of wards, and not provided for over 90 % of healthcare assistants. Over 60 % of nursing staff did not have access to clinical supervision, and 84 % of wards reported that no staff had access to guidance and support from a dementia champion with specialised knowledge and skills regarding best practice in the provision of dementia care. Finally, staff on the majority of the wards had no access to peer support groups (88 %) or reflective practice groups (95 %).

### Discharge planning and discharge

Discharge planning was not initiated within 24 h of admission for 72.2 % of patients, and only 39.1 % had a single plan for discharge with clear updated information in their chart. Only 30 % of patients had a named person co-ordinating their discharge. In the discharge summary, 32.5 % of patients had their support needs identified, 9.8 % had their level of cognitive impairment noted, and 34.9 % had the cause of impairment noted. Of those that had symptoms of delirium during the admission, 24.2 % had them summarised in their discharge summary, and of those who had symptoms of BPSD, 26.6 % had them summarised.

Carers needs (32.9 %) were infrequently assessed in advance of discharge. The appropriate place of discharge was discussed with 20.9 % of patients and 50.3 % of carers/relatives. Less than half of carers/relatives (41.3 %) received 24 h or more notice of discharge and only 11.6 % were given a copy of the patients’ discharge summary.

## Discussion

The objective of this audit was to investigate, for the first time, the quality of dementia care across all 35 acute public hospitals in the Republic of Ireland.

Generally, high levels of physical assessments were carried out, echoing findings relating to hospital dementia care in England and Wales [15, 16, 24] particularly for mobility, continence needs, and pressure sore risk; however a standardised assessment of functioning was carried out on only 36 % of patients with dementia. Assessing for changes in functional ability is important for prognostication, minimising hospital associated disability, and predicting mortality and other health outcomes during and after hospitalization [25], as well as for determining dependency to secure an adequate care package after discharge. All older patients, particularly those with dementia, should receive a standardized assessment of their functional ability during hospital admission.

Assessment for the presence of pain was poor, with almost one-quarter of patients not being asked about the presence of pain, or receiving any formal assessment. This reflects previous findings that pain is under-assessed in people with dementia [15, 24, 26]. Pain is common in older people with dementia, with up to 45 % experiencing pain [27]. While patients with dementia may not always be capable of communicating pain through self-report, pain can be assessed with tools through caregiver report and direct observation [28]. There are a number of validated tools that assess for indicators of pain, including the Pain Assessment in Advanced Dementia Scale [29]; such tools should be used in cases where verbal communication is not possible.

Mental status assessment was poor, which other research has found in this population [24] in a sample of 7934 people. Despite the fact that this sample had a known diagnosis of dementia, just over 40 % had any assessment of cognition, reflecting other findings in Ireland (55 %) [30], and in England and Wales (43 %) [15]. Cognitive assessment is vital in people with dementia to track course and prognosis, and determine the supports needed to manage the condition [31]. This population are also at high risk of developing delirium [32], however less than 30 % of patients were screened during their admission. Poor hospital delirium screening in dementia has been reported in other countries [16, 33–35]. Delirium superimposed on dementia is related to adverse outcomes including increased functional dependence, institutionalisation and mortality [36]. According to NICE guidelines [37], screening for fluctuations in behaviour should be carried out at least daily in the acute setting. The absence of this in over 70 % of charts reflects particularly poor practice in the care of a population at high risk of delirium onset.

Mood and BPSD were only assessed in 14 % of patients. Under-recognition and assessment of BPSD is common [38]. BPSD, including depression, are experienced by 66-97 % of people with dementia during the course of their condition [39]. Undetected and untreated symptoms can lead to significant caregiver burden [40] and consequently, earlier institutionalisation. Finally, less than half of patients had any collateral history recorded in their charts. This is imperative for the comprehensive assessment of change from baseline across a number of domains [41] and is central to treatment and prognostication.

Regarding hospital size, operationalised by bed capacity, the largest hospitals were more likely to carry out delirium screening and BPSD and mood assessments, and record collateral history, than the smallest hospitals. However, the largest hospitals were poorer at carrying out assessments of functioning and nutrition; the smallest hospitals these assessments more frequently. Cognitive assessments were completed most frequently in smaller hospitals. While these findings indicate important trends in dementia care by hospital size, bed capacity provides limited information for service planners about how to improve the quality of dementia care, as this approach does not account for organisational factors including the individual services provided within hospitals or the flexibility of staffing based on fluctuating demand [42, 43]. This is highlighted here by the large variation in assessments completed between the hospitals within each hospital size group, where resources would be expected to be reasonably similar.

There was a disparity between hospital policy regarding assessment for cognition, functional ability, nutritional status and BMI, and actual assessment in practice. In order to improve multidisciplinary assessment in hospital dementia care, better governance must happen at the hospital level; policies and guidelines must be put in place and enforced in practice at ward level. It is important that this issue of under-assessment, particularly mental assessments, for people with dementia is addressed in Irish hospitals; poor quality acute care has been suggested as contributing to an increased risk of adverse outcomes for older adults including those with dementia [44] Furthermore, those with dementia have an increased cost of care when admitted to hospital, due to greater lengths of stay compared to those without dementia [45]; improvements in multidisciplinary assessments to detect physical and mental problems would likely reduce the length of stay and consequently, cost of care.

According to ward managers, there was very poor access to psychology, continence services and social work, with 91, 66, and 47 % of wards reporting no access to these services respectively. People with dementia can have complex physical and psychosocial needs, particularly during a distressing hospital admission, and these services are vital to providing best care. Of note, there was some disagreement between wards in the same hospital about access to the above services, 9 % of wards disagreed about the availability of psychology and social work within the same hospital, and 17 % disagreed about continence services. This lack of clarity about the availability of services is reflective of fragmentation and a lack of coordination between different levels and settings of care [46]. Better communication is needed between hospital level and ward level management about which services are available within their hospitals. Much better access to pharmacy, physiotherapy, dietetics, speech and language therapy, occupational therapy, liaison psychiatry, and palliative care was reported. Future research should investigate the impact of specialist service availability in acute hospitals on the care and health outcomes of people with dementia, as well as the issue of fragmentation in relation to specialist services.

The provision of dementia awareness training is poor across hospitals; only two hospitals included it in their induction programmes. None of the 35 hospitals had mandatory training for any staff in dementia awareness, indicating that it is not currently considered a priority. Just over half of hospitals had dementia awareness training available to their nurses and doctors over the previous 12 months. However, almost two-thirds of ward managers reported that staff cover could not be provided for training relating to dementia care, indicating that staffing levels and lack of resources may be a barrier to staff attending any available training. This picture of inadequate dementia training provision for healthcare staff in acute settings has been found elsewhere also [15, 47]. Dementia awareness training should be mandatory for all staff that care for people with dementia in Ireland as it is integral to increasing overall quality of care and reducing staff burden.

Almost 70 % of wards had at least one vacancy between their permanent healthcare and nursing staff, and the mean number of nursing vacancies was 1.83, with a maximum of nine nursing vacancies. Lower staffing levels in hospital have been associated with reports of poorer quality care and nurse burnout, as well as adverse patient outcomes including higher mortality [48]. Low staffing levels arise from systemic problems, however in an effort to improve services for dementia, more resources will need to be allocated to ensure sufficient nurse-patient staffing ratios in hospitals.

There are very few supports in place for staff working with people with dementia, with mentorship and clinical supervision not available on 78 and 56 % of wards respectively. Further, peer support groups or reflective practice groups were not available to staff on 88 and 95 % of wards, and 84 % had no dementia champion to provide guidance on issues relating to dementia care. Dementia champion training is now available in Ireland, and so the number of hospitals with dementia champions may increase significantly in the near future. Perceived support from co-workers is important, and has been shown to enhance job performance and decrease the job stress among nursing staff [49]. It is important that management take responsibility for putting supports in place for staff caring for people with dementia to prevent burnout.

Discharge planning was quite poor; the majority of planning (72 %) was not initiated within 24 h of admission, and only 39 % of patients had a single detailed plan for discharge in their charts. The literature indicates that good discharge planning is imperative; inefficient planning is associated with adverse patient outcomes, increased length of stay, re-admission, and cost [22, 50].

Discharge destination was linked to LOS in this sample; the average LOS was 24.73 days, however further investigation revealed that those newly discharged to residential care had a significantly greater LOS than those not requiring such placement. The discharge of 35 % of people with dementia originally admitted from home to long term care in this sample is high compared to 11.5 % of older people without dementia in a recent study also carried out in 5 acute public hospitals [7]. This finding indicates that acute hospitals may be serving as an access point for entry to long term care for people with dementia struggling in the community. Overall, these findings suggest that the system for planning discharge teamed with poor availability of suitable residential placement for people with dementia is problematic in acute hospitals in Ireland, and is contributing to an increased LOS and consequently, greater cost of dementia care.

The results also indicated poor summarising of information, for example support needs, level/cause of cognitive impairment, and delirium and BPSD at discharge. This is poor practice, as the gap between hospital and community care is then not bridged and health outcomes cannot be effectively maintained or improved [22]. A more integrated approach to care across settings is required in order to reduce the unnecessary use of hospital beds once the patient is medically fit for discharge; this includes improved discharge planning, e.g. building relationships with post-acute care providers, and developing shared information systems to facilitate speedy transition [51].

Carer’s needs were found to be poorly assessed prior to discharge also, and they were found to have short notice of discharge. This is in line with reports that post-discharge, families feel ill-equipped to take on the carer role, and feel that staff neglected the component of discharge planning relating to accessing assistance and resources in the community [52]. Assessing carer support needs prior to discharge is important as caregiver burden is a risk for early institutionalisation [53].

This study has some limitations; the chart review data has been collected retrospectively, and these findings reflect what has been *recorded* in the charts. Theoretically, care relating to multidisciplinary assessment and discharge planning may have been better in practice; however any failure to record such information is a failure in communicating essential healthcare information to colleagues, which is, in and of itself, poor practice.

## Conclusion

Dementia care relating to assessment, access to certain specialist services, staffing levels, training and support, and discharge planning is suboptimal, which is likely to increase the risk of adverse patient outcomes and the cost of acute dementia care. Going forward, we must ensure that suitable pathways, policies and care practices are put in place to meet the complex needs of dementia patients to improve the overall quality of acute care provision.

## Abbreviations

BPSD, behavioural and psychological symptoms of dementia; LOS, length of stay; HIPE, hospital in-patient enquiry; HSE, Health Service Executive; IQR, interquartile range; BMI, body mass index.
